# Marginal grafts increase early mortality in liver transplantation

**DOI:** 10.1590/S1516-31802008000300005

**Published:** 2008-05-01

**Authors:** Telesforo Bacchella, Flávio Henrique Ferreira Galvão, José Luiz Jesus de Almeida, Estela Regina Figueira, Andreza de Moraes, Marcel Cerqueira César Machado

**Keywords:** Donor selection, Liver transplantation, Directed tissue donation, Tissue and organ procurement, Liver cirrhosis, Seleção do doador, Transplante de fígado, Doação dirigida de tecido, Obtenção de tecidos e órgãos, Cirrose hepática

## Abstract

**CONTEXT AND OBJECTIVE:**

Expanded donor criteria (marginal) grafts are an important solution for organ shortage. Nevertheless, they raise an ethical dilemma because they may increase the risk of transplant failure. This study compares the outcomes from marginal and non-marginal graft transplantation in 103 cases of liver transplantation due to chronic hepatic failure.

**DESIGN AND SETTING:**

One hundred and three consecutive liver transplantations to treat chronic liver disease performed in the Liver Transplantation Service of Hospital das Clínicas da Faculdade de Medicina da Universidade de São Paulo between January 2001 and March 2006 were retrospectively analyzed.

**METHODS:**

We estimated graft quality according to a validated scoring system. We assessed the pre-transplantation liver disease category using the Model for End-Stage Liver Disease (MELD), as low MELD (< 20) or high MELD (≥ 20). The parameters for marginal and non-marginal graft comparison were the one-week, one-month and one-year recipient survival rates, serum liver enzyme peak, post-transplantation hospital stay and incidence of surgical complications and retransplantation. The significance level was 0.05.

**RESULTS:**

There were no differences between the groups regarding post-transplantation hospital stay, serum liver enzyme levels and surgical complications. In contrast, marginal grafts decreased overall recipient survival one month after transplantation. Furthermore, low-MELD recipients of non-marginal grafts showed better one-week and one-month survival than did high-MELD recipients of marginal livers. After the first month, patient survival was comparable in all groups up to one year.

**CONCLUSION:**

The use of marginal graft increases early mortality in liver transplantation, particularly among high-MELD recipients.

## RESUMO

**CONTEXTO E OBJETIVO:**

A utilização de doadores com critérios expandidos (enxertos marginais) é uma importante solução para a carência de órgãos para transplante. No entanto, isto suscita importante dilema ético porque esses órgãos podem aumentar a chance de insucesso do transplante. Este estudo compara os resultados do transplante de órgãos marginais e não-marginais em 103 pacientes portadores de doença hepática crônica.

**TIPO DE ESTUDO E LOCAL:**

Cento e três transplantes consecutivos de fígado por doença hepática crônica realizados no Serviço de Transplante de Fígado do Hospital das Clínicas da Faculdade de Medicina da Universidade de São Paulo foram retrospectivamente analisados, compreendendo o período de janeiro de 2001 a março de 2006.

**MÉTODOS:**

A qualidade do enxerto foi calculada utilizando sistema de pontuação validado. A doença hepática do receptor também foi classificada em MELD (Model for End-Stage Liver Disease) baixo (< 20) e MELD alto (≥ 20). Os parâmetros avaliados na comparação entre receptores de órgãos marginais e não-marginais foram: sobrevida do paciente em uma semana, um mês e um ano, pico sérico das enzimas hepatocelulares, dias de internação pós-transplante e incidência de complicações cirúrgicas e retransplantes. O índice de significância foi de 0.05.

**RESULTADOS:**

Não houve diferença entre os grupos quanto ao tempo de internação pós-transplante, pico sérico das enzimas hepatocelulares e incidência de complicações cirúrgicas. Em contraste, a utilização de enxertos marginais diminuiu a sobrevida global de um mês. Os receptores de enxertos não-marginais com baixo MELD apresentaram melhor sobrevida em uma semana e um mês do que os receptores de enxertos marginais com alto MELD. Após o período de um mês, a taxa de sobrevida foi similar até um ano em todos os grupos.

**CONCLUSÃO:**

Em conclusão, o uso de enxerto marginal aumenta mortalidade precoce no transplante de fígado, principalmente nos receptores com alto MELD.

## INTRODUCTION

Organ shortage is a major problem in liver transplantation. The use of expanded donor criteria (marginal) grafts is an option used worldwide to increase the organ supply for transplantation. Marginal grafts are defined as those that involve the use of organs recovered from elderly donors (> 60 years old) or that present other harmful characteristics that may give rise to the danger that these allografts may lead to post-transplantation dysfunction or primary non-function. Nonetheless, the risk to recipients using such grafts remains imprecise and controversial.^1–13^

The guidelines for using marginal grafts vary from center to center. The pretransplantation liver disease status appears to be a valuable criterion for allocating these organs. Nevertheless, there is ongoing disagreement regarding this issue. Some authors have selected marginal grafts for healthier patients who can tolerate retransplantation, whereas others have advocated their unrestricted use to meet the needs of transplantation lists. Thus, there is a current debate about the regulation and results of marginal liver transplantation.^1–13^

## OBJECTIVE

The aim of this investigation was to compare the effects of marginal and non-marginal grafts for liver transplantation at the Liver Transplant Division, Hospital das Clínicas da Faculdade de Medicina da Universidade de São Paulo (HCFMUSP).

## MATERIALS AND METHODS

### Patients

In order to improve methodological accuracy, we included in this investigation 103 consecutive patients who had undergone their first liver transplantation between January 2001 and March 2006. Cases of transplantation for patients with acute liver failure, living donor transplantation, retransplantation and sequential transplantation were excluded. The patient characteristics and liver disease diagnoses are specified in Table 1. The procedure for liver graft recovery from deceased donors followed the protocol for our standard surgical technique.

**Table 1.** General characteristics and liver disease diagnoses of marginal and non-marginal graft recipients

	Non marginal	Marginal	p-value
**Gender**			
Male	26	39	
Female	12	26	
**Age (years)**	45.6 ± 2.3	45.3 ± 1.7	0.991
**Liver disease etiology**			
Virus B	7 (18.4%)	8 (12.3%)	0.401
Virus C	13 (34.2%)	26 (40%)	0.674
Alcoholic	6 (15.8%)	3 (4.6%)	0.072
Primary biliary cirrhosis	1 (2.6%)	4 (6.1%)	0.649
Cryptogenic	4 (10.5%)	4 (6.1%)	0.462
Autoimmune	4 (10.5%)	11 (16.9%)	0.563
Budd-Chiari	1 (2.6%)	1 (1.5%)	0.100
Familial amyloidotic polyneuropathy	2 (4.1%)	2 (3.1%)	0.624
Secondary biliary cirrhosis	0	1 (1.5%)	0.100
Alpha-1 antitrypsin deficiency	0	1 (1.5%)	0.100
Primary sclerosing cholangitis	0	4 (6.1%)	0.293

### Graft score system

We assessed graft condition quality using a previously described scoring system.^6,7^ Briefly, we gave a score of 1 for the following characteristics: donor > 60 years, orotracheal intubation period > 4 days, cold ischemia time > 13 hours, hepatic macrosteatosis ≥ 30%, bilirubin > 2.0 mg/dl, alanine aminotransferase (ALT) > 170 U/l and aspartate aminotransferase (AST) > 140 U/l. We gave a score of 2 to the following: use of vasopressor drugs (dopamine > 10 mg/kg or any dose of noradrenalin or dobutamine) and serum sodium > 155 mEq/l. We considered the liver to be marginal when the score reached 3 (i.e. ≥ 3).

### Pretransplantation patient status

We used the validated Model for End-Stage Liver Disease (MELD) scoring system to classify recipients before their transplantation, as low MELD (< 20) and high MELD (≥ 20).

### Experimental groups

We distributed recipients into six groups: Group 1 – All non-marginal liver recipients; Group 2 – All marginal liver recipients; Group 3 – Non-marginal livers for low-MELD recipients; Group 4 – Non-marginal livers for high-MELD recipients; Group 5 – Marginal livers for low-MELD recipients; Group 6 – Marginal livers for high-MELD recipients. We compared groups 1 and 2 independently from groups 3, 4, 5 and 6. The group distribution according to recipient MELD score and graft characteristics are presented in Table 2.

**Table 2.** Recipient distribution according to Model for End-stage Liver Disease (MELD) score and graft quality

	Marginal donors	Non-marginal donors	Total
MELD < 20	49 (47.6%)	30 (29.1%)	**79 (76.7%)**
MELD ≥ 20	16 (15.5%)	8 (7.8%)	**24 (23.3%)**
**Total**	**65 (63.1%)**	**38 (36.9%)**	**103 (100%)**

### Parameters of assessment

To compare marginal and non-marginal recipient outcomes, we evaluated the following parameters: serum peaks of aspartate aminotransferase (AST) and alanine aminotransferase (ALT), one-week, one-month and one-year recipient survival, incidence of retransplantation, length of post-transplantation hospital stay and incidence of surgical complications (Tables 3 and 4).

**Table 3.** Liver enzyme peak levels, length of hospital stay and overall one-month survival

	Marginal donors	Non-marginal donors	p-value
Serum peak of AST (U/l)	2394 ± 318	1529 ± 269	0.066
Serum peak of ALT (U/l)	1364 ±161	1154 ± 166	0.397
Hospital stay (days)	28 ± 3	28 ± 6	0.996
Overall one-month survival	52/65 (80.0%)	36/38 (94.7%)	0.046

AST = aspartate aminotransferase; ALT = alanine aminotransferase.

**Table 4.** Incidence of postoperative complications and retransplantation rate among recipients of marginal and non-marginal grafts

	Marginal donors	Non-marginal donors	Total	p-value
Abdominal collections	2 (3.1%)	0 (0%)	2	NS
Wound dehiscence	3 (4.6%)	1 (2.6%)	4	NS
Acute encephalopathy	1 (1.5%)	0 (0%)	1	NS
Portal vein thrombosis	0 (0%)	1 (2.6%)	1	NS
Evisceration	0 (0%)	1 (2.6%)	1	NS
Bleeding	1 (1.5%)	1 (2.6%)	2	NS
Pulmonary hypertension	2 (3.1%)	0 (0%)	2	NS
Acute renal failure	3 (4.6%)	2 (5.2%)	5	NS
Pontine myelinolysis	0 (0%)	1 (2.6%)	1	NS
Pancreatitis	1 (1.5%)	0 (0%)	1	NS
Hepatic artery thrombosis	4 (6.1%)	2 (5.2%)	6	NS
Hepatic vein thrombosis	1 (1.5%)	0 (0%)	1	NS
Retransplantation rate	7 (10.8%)	2 (5.2%)	9	NS

NS = non-significant.

### Immunosuppression

The immunosuppression was based on tacrolimus and corticosteroids. We weaned patients off corticosteroids within three months, excepted in cases of transplantation due to autoimmune hepatitis, primary biliary cirrhosis and primary sclerosis cholangitis, which were treated with corticosteroid continuously.

### Statistical analysis

We used the Kaplan-Meyer test to compare recipient survival, Fisher's exact test to assess the incidence of surgical complications and retransplantation, and Student's t test to evaluate the liver enzyme peak and length of post-transplant hospital stay. The significance level used was 0.05.

## RESULTS

**Figure 1:**
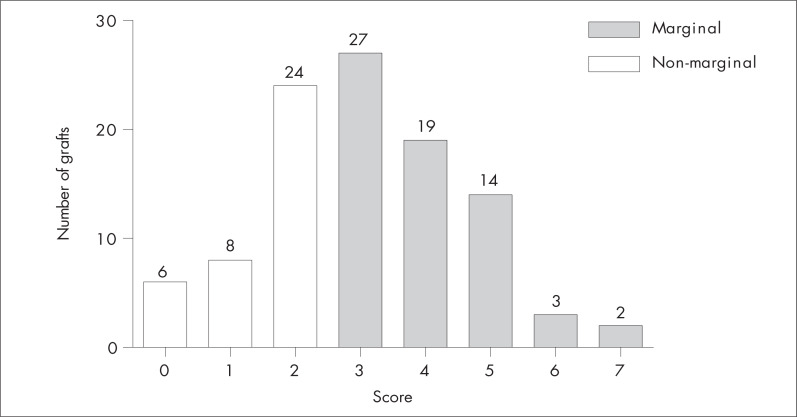
Graph showing liver quality distribution according to the score grading system of Figueira et al.^6^

**Figure 2:**
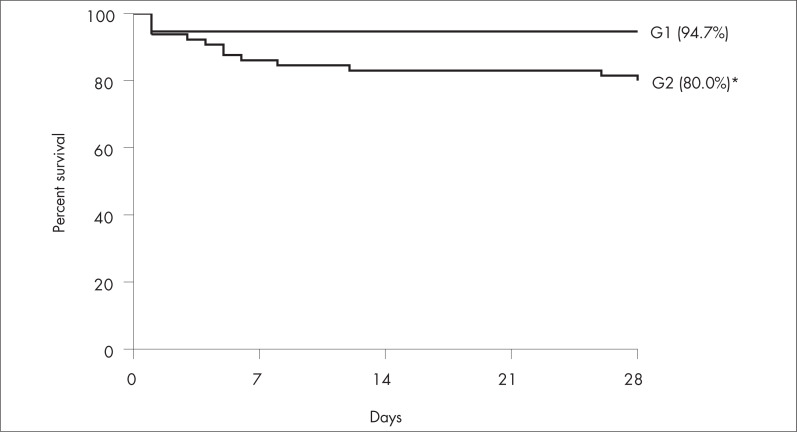
Graph showing overall four-week survival rates among non-marginal (G1) and marginal (G2) graft recipients (^*^p = 0.04).

**Figure 3:**
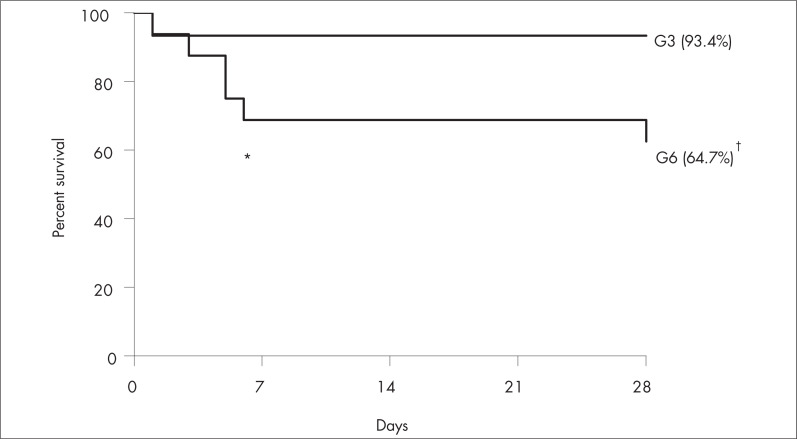
Graph showing survival rates among low-MELD (Model for End-stage Liver Disease) recipients of non-marginal grafts (G3) and high-MELD recipients of marginal grafts (G6) (one-week survival, ^*^p = 0.04; and four-week survival, ^†^p = 0.01).

Sixty-five grafts (63.1%) were considered marginal and 38 (36.9%) were considered non-marginal (Figure 1). Overall, the non-marginal graft recipients achieved a one-month survival rate of 94.7%, whereas the marginal graft recipients achieved 80.0% (Figure 2). Low-MELD recipients with non-marginal grafts achieved better survival rates after one week (93.4%) and one month (93.4%) than did high-MELD recipients with marginal livers (76.5% after one week and 64.7% after one month) (Figure 3). There were no differences in survival rate between marginal and non-marginal grafts for the other groups after one week, one month and one year. Therefore, there was no difference in survival between marginal and non-marginal grafts at times beyond the first month after transplantation. This finding suggests that marginal grafts increase the mortality rate only during the first month after transplantation.

There were no differences between the marginal and non-marginal groups regarding the serum peaks of AST and ALT, incidence of retransplantation, length of post-transplant hospital stay and incidence of surgical complications (Tables 3 and 4).

## DISCUSSION

In this study, we observed higher incidence of marginal donors than in most other centers that have assessed this subject.^3,4,7^ This may be explained in two ways: differences in graft grading score systems and local deficiencies in donor assistance.

There is no consensus regarding a grading system for assessing liver graft quality. Since these grading systems vary from center to center, it is reasonable to assume that some cases were classified in different categories depending on the system adopted. In the present report, we used a promising scoring system adapted from Briceño et al.^7^ that had been valuable in a previous investigation.^6^ Our scoring system was practical and offered a convenient assessment of liver quality at various degrees of marginal grading.

Concerning local deficiencies, it is difficult to compare Brazilian organ procurement organizations with other centers that have assessed marginal liver transplantation, which have usually been located in developed countries. In Brazil, organ donation and recovery indexes remain poor. According to the 2006 edition of the National Transplant Register (RNT) of the Brazilian Organ Transplantation Association (ABTO),^14^ there were only six effective donors per million people per year, out of 30.6 brain death notifications per million people per year in Brazil. Furthermore, many physicians ignore the legislation regarding organ donation and have misunderstandings about the diagnosis of brain death and maintenance of potential donors.^15,16^ The deficiencies in organ procurement and donor assistance in Brazil may influence graft quality, thereby causing high incidence of marginal liver use.

Differing from our results, previous studies have not found any differences in survival between the recipients of marginal and non-marginal grafts. Nonetheless, they have demonstrated higher incidence of primary non-function and retransplantation among the recipients of marginal grafts.^3–5,8–13^ This suggests that the high mortality rate among marginal graft recipients in the present report may relate to the prolonged time that elapses until retransplantation can be performed in patients with primary non-function. The long time it takes to find a suitable graft for these critical patients may negatively affect the survival rate.

Appropriate care for potential donors is a crucial step in increasing the numbers of organs available for transplantation. Continuing medical education on organ transplantation and public debates about this topic are important conditions for improving organ donation and the quality of the recovery grafts.^15–17^ Physicians’ training in potential donor management allows application of methods including hormone replacement in order to avoid rapid donor deterioration.^18^ Furthermore, the release of accurate information to the population about brain death and organ recovery following brain death affects decisions on whether to authorize organ donation.^17^

In this report, we observed that the first month after transplantation was decisive for defining the overall marginal graft outcome and recipient survival. In addition, high-MELD recipients of marginal grafts suffered high mortality within the first week after transplantation. This mortality would have been even higher if it had not been possible to perform retransplantation in some patients in this trial. Thus, if primary non-function occurs, prompt retransplantation is critical to save the recipient's life. Unfortunately, it is impossible to predict accurately which grafts will fail during the early post-transplantation period. Beyond the first month after transplantation, the recipients of marginal and non-marginal grafts achieved similar outcomes.

The ideal match between recipient clinical status and degree of graft injury is an important issue in liver transplantation. We observed high mortality within the first month in cases of marginal grafts transplanted into high-MELD recipients. On the other hand, high-MELD recipients of non-marginal livers achieved an adequate survival rate. Thus, this raises the question of which patients should be eligible to receive a marginal liver.

Melendez and Heaton^2^ pointed towards the idea that marginal grafts should be used only in clinically stable patients who can tolerate the possibility of retransplantation. Similarly, Cameron et al.^13^ established new criteria for using marginal livers and matched such livers with younger patients with initial cirrhosis and hepatocellular cancer. In order to expand the donor pool, they used marginal organs only in low-MELD recipients, and non-marginal ones in high-risk patients (high-MELD).

In contrast, Pokorny et al.^4^ stated that refusal to use marginal grafts would decrease transplantation activity, thereby increasing mortality on the waiting list. These authors accepted any marginal graft and managed any early graft dysfunction by means of aggressive retransplantation. This practice raises ethical controversy because retransplantation increases recipient morbidity-mortality. In addition, it is very difficult to predict when a suitable graft will become available for urgent retransplantation in Brazil. This situation may increase early mortality, thus impeding patients’ chances of obtaining suitable grafts.

With the aim of improving the liver transplantation system, a recent Brazilian federal law (Federal Decree no. 1160, May 29, 2006) established liver graft allocations based on patients’ liver disease severity instead of the usual chronological system, thus following the international trend. In this system, patients with higher MELD scores have priority in receiving liver grafts. Because of complex local situations regarding organ procurement, transplantation of marginal grafts into high-MELD recipients is nowadays a frequent occurrence in Brazilian centers.

Our Liver Transplantation Division has one of the largest waiting lists for liver transplantation in Brazil, with more than 800 patients. Rational use of marginal graft allocation is frequently debated in our meetings. We have established a limit for transplanting these marginal livers. We do not proceed with transplanting any graft if the donor attains one of the following conditions: serum sodium > 180 mEq/l, creatinine > 3.0 mg/dl, AST and/or ALT > 600 U/l, bilirubin > 3.0 mg/dl, donor age > 75 years and body weight < 30 kg or > 100 kg. We are currently analyzing our results from liver transplantations carried out over the first year of this new allocation system in Brazil.

Recently implemented pretransplantation therapeutic methods have had the aim of compensating for the inconvenience of organ shortage. Protocols combining refinements of liver surgery techniques and immunotherapy can remove complex liver tumors, thus avoiding the requirement for liver transplantation.^19^ Furthermore, recent advances in biotechnology have made it possible to apply hepatocyte transplantation as a promising and less aggressive procedure for treating liver disease. This method has been useful for treating metabolic liver disease and may reduce liver transplantation waiting lists.^20^

## CONCLUSION

Marginal grafts increase early mortality in liver transplantation cases, and particularly among high-MELD patients.
